# Tirzepatide for Weight Recurrence After Metabolic and Bariatric Surgery: A Critical Review of Direct and Indirect Evidence with Emphasis on Sleeve Gastrectomy, Nutritional Safety, and Clinical Implications

**DOI:** 10.3390/nu18193131

**Published:** 2026-09-23

**Authors:** Karolina Ciołkosz, Ewelina Polak-Szczybyło

**Affiliations:** 1 Student Scientific Club of Human Nutrition, Faculty of Health Sciences and Psychology, Collegium Medicum, University of Rzeszów, ul. Warzywna 1a, 35-959 Rzeszow, Poland; kc127395@stud.ur.edu.pl; 2 Department of Dietetics, Faculty of Health Sciences and Psychology, Collegium Medicum, University of Rzeszów, ul. Warzywna 1a, 35-959 Rzeszow, Poland

**Keywords:** obesity, sleeve gastrectomy, metabolic and bariatric surgery, tirzepatide, weight recurrence, body composition, nutritional status, dietetic management

## Abstract

**Background:** Obesity is a chronic, relapsing disease associated with substantial morbidity and healthcare burden. Although metabolic and bariatric surgery (MBS) remains the most effective treatment for severe obesity, weight recurrence after sleeve gastrectomy (SG) represents a significant clinical challenge and may require additional therapeutic strategies. Tirzepatide, a dual glucose-dependent insulinotropic polypeptide (GIP) and glucagon-like peptide-1 (GLP-1) receptor agonist, has demonstrated substantial efficacy in obesity treatment; however, evidence regarding its use after SG remains limited. This review critically evaluates the current evidence regarding the potential role of tirzepatide in the management of weight recurrence after SG, with particular emphasis on treatment efficacy, safety, body composition, nutritional considerations, and implications for dietetic practice. **Methods:** A critical review of the literature was conducted using PubMed/MEDLINE, Scopus, Web of Science, Cochrane Library, and ClinicalTrials.gov databases. Evidence was analyzed according to the population studied, including direct evidence from patients after SG, data from mixed post-bariatric surgery cohorts, and indirect evidence from clinical trials involving individuals with obesity without previous MBS. **Results:** Available evidence suggests that tirzepatide may induce clinically meaningful weight reduction in patients experiencing weight recurrence after SG; however, direct evidence remains limited and is derived mainly from short-term retrospective studies. Findings from mixed post-bariatric populations and randomized clinical trials in individuals with obesity provide supportive but indirect evidence. Important uncertainties remain regarding long-term efficacy, treatment durability after discontinuation, effects on body composition, preservation of fat-free mass, and nutritional safety. **Conclusions:** Tirzepatide may represent a valuable adjunctive therapy for selected patients with weight recurrence after SG, particularly when integrated into multidisciplinary obesity management. Nevertheless, current evidence is insufficient to establish its definitive role after SG. Future prospective studies are warranted to evaluate long-term effectiveness, safety, nutritional outcomes, and changes in body composition using standardized definitions of postoperative weight recurrence.

## 1. Introduction

Weight recurrence following metabolic and bariatric surgery (MBS), particularly after sleeve gastrectomy (SG), the most commonly performed bariatric procedure worldwide, represents a major clinical challenge. It is associated with the recurrence or worsening of obesity-related comorbidities, deterioration in quality of life, and the need for additional therapeutic interventions, including anti-obesity pharmacotherapy or revisional bariatric surgery, such as conversion to Roux-en-Y gastric bypass (RYGB). However, revisional surgery is technically more demanding and carries a higher risk of complications than primary procedures. Consequently, pharmacotherapy is increasingly considered an attractive therapeutic option for patients experiencing weight recurrence after MBS [1,2]. Tirzepatide, a dual glucose-dependent insulinotropic polypeptide (GIP) and glucagon-like peptide-1 (GLP-1) receptor agonist, has emerged as a highly effective pharmacological treatment for obesity. Nevertheless, evidence regarding its use in patients following SG remains limited. The aim of this critical review is to evaluate the current evidence on the efficacy of tirzepatide for the management of weight recurrence after SG, with particular emphasis on treatment safety, body composition, nutritional aspects, and implications for dietetic practice. Available studies suggest that tirzepatide may induce clinically meaningful short-term weight loss in patients after MBS and, in some observational studies, has demonstrated greater effectiveness than semaglutide. However, the current evidence is derived primarily from retrospective studies with short follow-up periods and is limited by heterogeneous definitions of weight recurrence, small sample sizes, and inadequate reporting of nutritional outcomes [3,4]. Important unanswered questions remain regarding the effects of tirzepatide on nutrient intake, particularly protein intake, food tolerance, preservation of fat-free mass, the risk of sarcopenia, and the long-term sustainability of treatment outcomes following therapy discontinuation [5,6,7,8,9].

Although SG represents one of the most commonly performed metabolic and bariatric procedures, evidence regarding pharmacological management of weight recurrence after SG remains limited. Therefore, interpretation of available data should consider both SG-specific studies and indirect evidence derived from mixed post-bariatric populations and clinical trials conducted in individuals with obesity without previous surgery.

## 2. Methods

This study was conducted as a critical review of the literature. A comprehensive search of the PubMed/MEDLINE, Scopus, Web of Science, Cochrane Library, and ClinicalTrials.gov databases was performed to identify publications evaluating the use of tirzepatide for the management of weight recurrence after metabolic and bariatric surgery (MBS), with particular emphasis on patients who had undergone sleeve gastrectomy (SG).

The final literature search was conducted in July 2026. Publications published between 2000 and 2026 were considered. The primary focus was placed on recent evidence, particularly studies published within the last eight years, reflecting current developments in obesity pharmacotherapy and incretin-based therapies. Older publications were included when they provided essential background information regarding hormonal, metabolic, anatomical, or nutritional consequences of bariatric surgery.

The search strategy included the following keywords and their combinations: “tirzepatide,” “sleeve gastrectomy,” “bariatric surgery,” “weight recurrence,” “weight regain,” “Roux-en-Y gastric bypass,” “anti-obesity medication,” “GLP-1 receptor agonist,” “GIP/GLP-1 receptor agonist,” “protein intake,” “nutritional deficiencies,” “body composition,” and “fat-free mass.”

Eligible publications included clinical trials, observational studies, systematic reviews, meta-analyses, clinical practice guidelines, and expert consensus statements published in English. Studies were considered eligible when they provided relevant information regarding tirzepatide efficacy or safety, pharmacological management after metabolic and bariatric surgery, postoperative weight recurrence or insufficient weight loss after sleeve gastrectomy or Roux-en-Y gastric bypass (RYGB), nutritional outcomes following bariatric surgery, or body composition changes associated with anti-obesity pharmacotherapy. Studies were excluded when they did not provide clinically relevant information regarding postoperative weight management, obesity pharmacotherapy, nutritional safety, or body composition outcomes.

Studies addressing weight recurrence after SG or RYGB, pharmacotherapy following MBS, the efficacy of tirzepatide in obesity treatment, body composition, nutritional status, nutrition-related deficiencies, and treatment safety were considered. Due to the limited number of studies directly evaluating tirzepatide after SG, evidence from individuals with obesity without previous bariatric surgery was also considered when relevant; however, these data were interpreted as indirect evidence and were not regarded as SG-specific evidence.

The core body of the literature consisted of the most recent publications from the last eight years, allowing for the inclusion of up-to-date data on obesity pharmacotherapy, tirzepatide, weight recurrence after MBS, and nutritional safety. Selected older publications, including studies from 2000 and 2007–2010, were retained only when they were considered foundational or necessary for understanding hormonal, metabolic, and nutritional mechanisms following bariatric surgery.

The literature was critically appraised with a particular focus on treatment efficacy, safety, methodological limitations of the available evidence, and current controversies regarding protein intake, nutritional deficiencies, food tolerance, changes in body composition, preservation of fat-free mass, and the potential risk of sarcopenia.

Because direct evidence regarding tirzepatide after SG remains limited, the available literature was interpreted with consideration of the strength and applicability of evidence. Studies were discussed according to whether they provided SG-specific evidence, evidence from heterogeneous post-bariatric populations involving different surgical procedures, or indirect evidence from obesity trials conducted in individuals without previous bariatric surgery.

## 3. Obesity: Current Concepts and Treatment Strategies

Contemporary concepts of obesity have moved beyond defining the disease solely on the basis of body mass index (BMI). Obesity is now recognized as a chronic, relapsing, multifactorial disease resulting from complex interactions among metabolic, hormonal, genetic, environmental, and behavioral factors. Increasing evidence indicates that excessive weight gain is not merely a consequence of inadequate self-control but reflects dysregulation of the homeostatic mechanisms governing appetite, energy expenditure, and adipose tissue storage.

The recent *Lancet Diabetes & Endocrinology* Commission proposed a new framework for defining obesity, emphasizing that obesity should not be characterized solely by BMI but by excess or abnormal adipose tissue accumulation and its consequences for organ function and daily living. The Commission further distinguished “preclinical obesity”, characterized by excess adiposity without evidence of organ dysfunction, from “clinical obesity”, in which excess adiposity is already associated with organ impairment, clinical manifestations, or functional limitations [10].

Obesity substantially increases the risk of numerous chronic diseases, including type 2 diabetes, dyslipidemia, cardiovascular disease, metabolic dysfunction-associated steatotic liver disease, and obstructive sleep apnea. Although BMI and waist circumference remain the most widely used measures in routine clinical practice, they do not adequately reflect body fat distribution or individual metabolic risk. This concept highlights that the primary goal of obesity treatment extends beyond weight reduction alone, encompassing improvements in metabolic health, physical function, and quality of life. Consequently, weight recurrence following obesity treatment may represent more than a simple increase in body weight and could reflect a potential relapse of a chronic disease requiring comprehensive long-term management [10,11,12].

The global burden of obesity continues to rise. Between 1990 and 2021, the prevalence of overweight and obesity increased substantially worldwide. In 2021, approximately 1.00 billion adult men and 1.11 billion adult women were living with overweight or obesity. If current trends continue, this number is projected to reach approximately 3.8 billion adults by 2050, representing more than half of the global adult population. These projections underscore the urgent need for effective long-term prevention and treatment strategies [13].

The management of obesity is generally considered a comprehensive, lifelong process combining lifestyle modification, dietary intervention, physical activity, pharmacotherapy, and, when indicated, metabolic and bariatric surgery (MBS). According to the current guidelines of the American Society for Metabolic and Bariatric Surgery (ASMBS) and the International Federation for the Surgery of Obesity and Metabolic Disorders (IFSO), MBS is recommended for individuals with a BMI ≥ 35 kg/m^2^ regardless of obesity-related comorbidities. Surgery may also be considered for patients with a BMI of 30.0–34.9 kg/m^2^ and metabolic disease when nonsurgical treatment fails to achieve durable weight loss or meaningful clinical improvement [14]. Sleeve gastrectomy (SG) and Roux-en-Y gastric bypass (RYGB) are currently the two most commonly performed bariatric procedures worldwide. Randomized controlled trials have demonstrated that both procedures achieve substantial and durable weight loss while improving obesity-related comorbidities. The SM-BOSS trial reported comparable reductions in BMI five years after surgery, although remission of gastroesophageal reflux disease was more frequent following RYGB. Similarly, the SLEEVEPASS trial demonstrated slightly greater weight loss after RYGB at seven years; however, the difference was not considered clinically meaningful, and quality of life was comparable between procedures. Ten-year follow-up data from the SM-BOSS trial further confirmed the long-term effectiveness of both techniques while suggesting a possible advantage of RYGB with respect to reflux control and weight loss in per-protocol analyses. Nevertheless, a considerable proportion of patients experience insufficient weight loss or subsequent weight recurrence, highlighting the need for long-term follow-up and effective adjunctive therapeutic strategies after bariatric surgery [14,15,16,17]. Therefore, despite the high effectiveness of MBS, the chronic nature of obesity means that some patients may require additional therapeutic strategies after surgery, including pharmacological interventions targeting appetite regulation and metabolic control.

## 4. Weight Recurrence and Inadequate Weight Loss After Metabolic and Bariatric Surgery

Despite the high efficacy of MBS, both weight recurrence and inadequate weight loss remain important clinical challenges following SG and RYGB. Interpretation of the reported prevalence of postoperative weight recurrence is complicated by the lack of a universally accepted definition of this outcome [1,18]. In a systematic review involving patients who underwent SG, the prevalence of weight recurrence ranged from 5.7% at 2 years to 75.6% after 6 years of follow-up [19]. Similarly, a comparative analysis of SG and RYGB demonstrated that even among patients who initially achieved ≥20% total weight loss (TWL) one year after surgery, return to baseline body weight occurred more frequently after SG than after RYGB (22.1% vs. 12.7%, respectively) [2,20]. Akpinar et al. further demonstrated that, among patients who achieved at least 20% TWL during the first postoperative year, weight recurrence between the second and fifth postoperative years occurred more frequently after SG than after RYGB. Although both groups initially responded well to surgery, weight recurrence between the second and fifth postoperative years was significantly more common after SG. Moreover, patients undergoing SG were less likely to maintain ≥20% TWL and less frequently achieved remission of obesity-related comorbidities. These findings suggest that the assessment of postoperative weight recurrence may benefit from a broader perspective, considering not only the magnitude of weight regain from the weight nadir but also the maintenance of total weight loss and the recurrence or persistence of obesity-related diseases [2]. Collectively, these findings emphasize the importance of long-term follow-up and early identification of postoperative weight recurrence, even in patients who initially achieve satisfactory weight loss. Nevertheless, the available evidence warrants cautious interpretation due to substantial heterogeneity in the definitions of weight recurrence, follow-up duration, and patient characteristics across studies.

### 4.1. Causes of Weight Recurrence After Metabolic and Bariatric Surgery

Weight recurrence after MBS is a multifactorial phenomenon resulting from the interplay of anatomical, metabolic, hormonal, behavioral, and psychological factors. Following SG, contributing factors may include an excessively large initial gastric sleeve, progressive sleeve dilation, incomplete fundus resection, increased gastric capacity over time, and alterations in appetite and satiety regulation. After RYGB, enlargement of the gastrojejunal anastomosis or other anatomical changes that reduce the restrictive and metabolic effects of surgery may contribute to postoperative weight recurrence. Regardless of the surgical procedure, behavioral and lifestyle factors also play a crucial role. Increased energy intake, frequent consumption of energy-dense foods, grazing behavior, intake of liquid calories, insufficient physical activity, eating disorders, psychological factors, and inadequate long-term postoperative follow-up may contribute to increased risk of postoperative weight recurrence [1,19]. Lauti et al. additionally highlighted potential hormonal mechanisms, including changes in circulating ghrelin concentrations, together with anatomical factors and insufficient postoperative support, as contributors to weight recurrence after SG [20].

Weight recurrence following bariatric surgery represents a clinically significant challenge because it may compromise the long-term benefits of surgical treatment. Although MBS generally results in substantial weight loss and improvement or remission of obesity-related comorbidities, these effects are not always durable. Subsequent weight recurrence may be accompanied by deterioration of metabolic homeostasis and recurrence or progression of type 2 diabetes, hypertension, dyslipidemia, obstructive sleep apnea, and other obesity-related complications. Therefore, weight recurrence may be viewed not merely as an anthropometric change but rather as a potential manifestation of the relapsing nature of obesity and its associated clinical consequences [1,2,19]. King et al. demonstrated that the method used to quantify postoperative weight recurrence substantially influences its association with clinical outcomes. Among the evaluated metrics, the percentage of maximum weight lost that was subsequently regained emerged as a potentially relevant indicator associated with adverse clinical outcomes. Consequently, postoperative assessment may require a broader evaluation that considers current BMI or body weight alongside the patient’s weight trajectory, weight nadir, rate of subsequent weight gain, and recurrence of obesity-related comorbidities. From this perspective, weight recurrence after SG or RYGB may be considered a manifestation of recurrent chronic obesity that warrants comprehensive reassessment and individualized therapeutic management [21]. From a clinical perspective, postoperative weight recurrence frequently necessitates a comprehensive diagnostic evaluation. In patients following SG or RYGB, this may include assessment of anatomical factors, such as sleeve dilation, increased gastric volume, enlargement of the gastrojejunal anastomosis after RYGB, or other structural abnormalities compromising surgical efficacy. At the same time, behavioral, psychological, dietary, and metabolic factors—including dietary habits, energy intake, grazing behavior, consumption of liquid calories, physical activity, eating disorders, and adherence to long-term follow-up—may be considered important components of postoperative assessment. Importantly, postoperative weight recurrence may be better understood not as simple “patient failure” but as a complex clinical condition influenced by multiple factors and potentially requiring multidisciplinary management [1,19].

Management may therefore require intensification of treatment through renewed dietary intervention, psychological support, anti-obesity pharmacotherapy, endoscopic procedures, or revisional bariatric surgery. This is particularly relevant after SG, as several studies have reported a higher risk of weight recurrence compared with RYGB [2]. These findings indicate that even patients who initially achieve satisfactory postoperative weight loss require long-term follow-up and ongoing therapeutic support.

### 4.2. Definitions of Weight Recurrence After Metabolic and Bariatric Surgery

One of the major limitations of the current literature on postoperative weight outcomes following MBS is the lack of standardized terminology. Contemporary evidence highlights the need to distinguish between several distinct postoperative weight outcomes, including *weight recurrence*, *partial response*, and *nonresponse* to surgical treatment. El Ansari and Elhag emphasized that numerous inconsistent definitions of *weight regain* and *insufficient weight loss* have been used across studies, making it difficult to compare prevalence estimates and evaluate the effectiveness of therapeutic interventions [1]. King et al. compared several methods for defining and quantifying postoperative weight regain and demonstrated that the reported prevalence varied substantially depending on the definition and threshold applied. Among the evaluated metrics, the percentage of maximum weight loss regained demonstrated the most consistent association with clinically relevant outcomes [21]. Similarly, Voorwinde et al. evaluated six different definitions of long-term weight regain in patients five years after RYGB or SG and examined their association with clinical outcomes. The prevalence of weight regain varied considerably according to the definition used. Although some degree of weight regain was observed in the majority of patients, the proportion meeting criteria for clinically significant weight regain ranged from 16% to 37%. Importantly, only selected definitions were associated with deterioration in the physical component of quality of life, whereas no statistically significant associations were observed between most definitions of weight regain and obesity-related comorbidities. These findings emphasize the importance of clearly reporting the definition of postoperative weight recurrence, the reference point used for its calculation, and the clinical relevance of the observed weight change, particularly in studies evaluating pharmacological treatment after SG [22]. To address these inconsistencies, the POWER Task Force of the ASMBS proposed updated terminology for describing postoperative weight outcomes. Majid et al. suggested replacing the terms *weight regain* and *recidivism* with *weight recurrence*, as the latter more accurately reflects the chronic, relapsing nature of obesity while avoiding language that may imply patient blame. Likewise, the authors suggested using the term *nonresponder* instead of *inadequate weight loss* to describe patients who fail to achieve an adequate postoperative weight reduction [18]. The same expert group proposed consensus-based definitions intended to improve terminology and reporting consistency. *Partial response* was proposed as achieving 10–20% total weight loss (TWL), *nonresponse* was proposed as <10% TWL, and *clinically significant weight recurrence* was defined as regaining >20% of the maximum weight previously lost [23]. Adoption of standardized terminology is essential for improving comparability across studies and facilitating the interpretation of treatment outcomes in both clinical practice and research. Weight recurrence and insufficient weight loss remain major barriers to the long-term effectiveness of metabolic and bariatric surgery. Establishing a universally accepted, clinically meaningful definition of postoperative weight outcomes is essential for both research and clinical practice. In the context of pharmacological management after bariatric surgery, it is particularly important to specify whether treatment is being initiated for *weight recurrence*, *insufficient weight loss (nonresponse),* or the broader entity of *suboptimal clinical response*, as these clinical scenarios differ substantially in their underlying mechanisms, prognosis, and therapeutic approach. Given the lack of standardized definitions of postoperative weight outcomes, the terminology and outcome measures used throughout this review are summarized in Table 1.

## 5. Tirzepatide as Adjunctive Therapy After Metabolic and Bariatric Surgery

Tirzepatide has emerged as a novel pharmacological option within the expanding therapeutic landscape of obesity management, including potential use as an adjunctive therapy after metabolic and bariatric surgery. A considerable proportion of patients undergoing SG or RYGB experience either inadequate weight loss or subsequent weight recurrence. Consequently, anti-obesity pharmacotherapy has gained increasing recognition as an adjunct to bariatric surgery, particularly when integrated with dietary and behavioral interventions [23,24]. Pharmacological treatment has increasingly been considered as part of the multimodal management of postoperative weight recurrence, particularly in patients who do not require or are not suitable for revisional procedures [24].

Tirzepatide is a dual glucose-dependent insulinotropic polypeptide (GIP) and glucagon-like peptide-1 (GLP-1) receptor agonist that exerts its effects by regulating appetite, satiety, glycemic control, and body weight. In the SURMOUNT-1 trial, tirzepatide produced substantial weight loss over 72 weeks in adults with overweight or obesity, significantly outperforming placebo [25]. Findings from the SURMOUNT-3 trial further demonstrated that tirzepatide enhances weight reduction following an initial lifestyle intervention, highlighting its role as part of a comprehensive obesity management strategy rather than a substitute for dietary therapy [26]. The clinical value of tirzepatide was further reinforced by the SURMOUNT-4 trial, which showed that continued treatment was associated with sustained weight loss, whereas treatment discontinuation resulted in significant weight regain [27].

### 5.1. Mechanism of Action of Tirzepatide

Tirzepatide is distinguished from selective GLP-1 receptor agonists, such as semaglutide and liraglutide, by its dual agonistic activity at both GIP and GLP-1 receptors. This dual mechanism is associated with several complementary effects relevant to obesity treatment, including: suppression of appetite, enhancement of satiety, indirect reduction in energy intake, improvement of glycemic control, reduction in body weight and adipose tissue mass [22,28].

The mechanism of action of tirzepatide is particularly relevant in the context of the biological adaptations that promote weight regain after weight loss. Following weight reduction, compensatory mechanisms—including increased appetite, enhanced hunger signaling, and reduced energy expenditure—favor the restoration of body weight. By activating both GIP and GLP-1 pathways, tirzepatide may influence some of these adaptive responses through effects on appetite regulation and postprandial glucose metabolism. Clinical trials have consistently demonstrated its efficacy in promoting clinically meaningful weight loss in individuals with obesity [22]. In patients undergoing SG, this mechanism is of both theoretical and clinical relevance. Tirzepatide may enhance incretin-mediated satiety signaling and attenuate the physiological mechanisms that contribute to postoperative weight recurrence. However, it is important to acknowledge that most mechanistic evidence has been derived from studies in individuals with obesity who had not previously undergone metabolic and bariatric surgery. Consequently, the relevance of these findings to patients after SG requires consideration of this limitation. Importantly, although the mechanism of action of tirzepatide provides a plausible biological rationale for its potential use following SG, mechanistic evidence alone may not fully address nutrition-related safety considerations. Marked appetite suppression and reduced energy intake may contribute to the prevention of weight recurrence; however, they may also be associated with an increased risk of inadequate protein intake, micronutrient deficiencies, food intolerance, and reductions in fat-free mass among patients after MBS. Therefore, the evaluation of tirzepatide after SG may benefit from extending beyond weight-loss efficacy alone and incorporating assessment of body composition, nutritional status, and long-term treatment safety [22,29,30].

### 5.2. Sleeve Gastrectomy and the Incretin Axis in the Context of Tirzepatide Therapy

A meta-analysis by McCarty et al. demonstrated that SG induces significant changes in the secretion of gastrointestinal hormones involved in the regulation of appetite, satiety, and glucose metabolism. Following SG, fasting ghrelin concentrations decrease, whereas postprandial secretion of GLP-1 and peptide YY (PYY) increases. In contrast, the effects of SG on GIP secretion remain less consistent. These findings indicate that the benefits of SG extend beyond mechanical gastric restriction and involve endocrine adaptations that contribute to appetite suppression, enhanced satiety, and improved metabolic control [31]. The primary sites of secretion, physiological functions, changes following SG, and potential clinical relevance of the principal gastrointestinal hormones are summarized in Table 2.

Following SG, hormonal adaptations include reduced ghrelin concentrations together with enhanced postprandial secretion of GLP-1 and PYY. Because incretin-related pathways remain biologically relevant after surgery, tirzepatide has a plausible mechanistic rationale as an adjunctive therapy in patients experiencing postoperative weight recurrence. Nevertheless, available evidence indicates that incretin-related mechanisms alone do not fully explain the metabolic benefits of SG. Hormonal responses vary considerably among individuals and may diminish over time, suggesting that enhancement of GLP-1 and GIP signaling alone is unlikely to completely prevent postoperative weight recurrence [30,31,37,38,39].

The rationale for using tirzepatide after SG is based on the concept that sustained postoperative weight loss depends not only on gastric restriction but also on long-term regulation of appetite, satiety, glycemic control, and metabolic adaptation. In some patients, the hormonal changes induced by SG may become insufficient to maintain weight loss, particularly in the presence of behavioral, metabolic, or anatomical factors promoting weight recurrence [1,20,31]. In this context, tirzepatide may serve as an adjunctive therapy by enhancing incretin-mediated regulation of appetite, satiety, glycemic control, and body weight. Therefore, its potential role after SG is not to replace the effects of surgery but rather to pharmacologically modulate physiological mechanisms that may become inadequate for maintaining long-term treatment success [22,38]. However, mechanistic plausibility does not replace clinical evidence, and the potential benefits of tirzepatide after SG require confirmation in well-designed clinical studies specifically involving patients with postoperative weight recurrence. Thus, the mechanistic rationale offers a plausible basis for further investigation of tirzepatide after SG, while currently serving primarily as a hypothesis-generating framework rather than evidence of confirmed clinical efficacy.

## 6. Evidence for Tirzepatide After Metabolic and Bariatric Surgery: Direct and Indirect Evidence with Emphasis on Sleeve Gastrectomy

Evidence regarding tirzepatide after metabolic and bariatric surgery needs to be considered alongside the broader evidence base available for obesity treatment. Although the efficacy of tirzepatide in individuals with overweight or obesity without previous bariatric surgery has been demonstrated in large randomized clinical trials, these findings represent indirect evidence for patients after bariatric surgery. In the SURMOUNT-1 trial, tirzepatide produced substantial, dose-dependent weight loss in adults with overweight or obesity [25]. However, these findings cannot be directly extrapolated to patients following sleeve gastrectomy (SG), owing to postoperative alterations in gastrointestinal anatomy, changes in nutrient tolerance, the risk of nutritional deficiencies, and differences in postoperative weight-loss dynamics.

### 6.1. Direct Evidence After Sleeve Gastrectomy

Current evidence regarding the use of tirzepatide for the management of weight recurrence after SG remains promising but limited. The most relevant study specifically addressing this population is the retrospective analysis by Jamal et al., which compared semaglutide and tirzepatide in patients experiencing weight recurrence following SG. After six months of treatment, both therapies resulted in significant weight loss; however, the reduction was significantly greater in the tirzepatide group. Mean weight loss reached 10.3% with semaglutide compared with 15.5% with tirzepatide, suggesting that tirzepatide may represent a potentially effective short-term adjunctive option in selected patients with postoperative weight recurrence [3]. Nevertheless, several important limitations need to be considered. The study was retrospective, included a relatively small number of patients receiving tirzepatide, had a short follow-up period, and did not comprehensively evaluate nutritional outcomes. Protein intake, micronutrient deficiencies, food tolerance, body composition, and weight changes following treatment discontinuation were not systematically assessed. Consequently, the findings may be considered an important proof-of-concept, while further evidence is needed to establish the long-term efficacy and safety of tirzepatide after SG [3].

### 6.2. Evidence from Mixed Post-Bariatric Populations

Because SG-specific evidence remains scarce, studies involving heterogeneous post-bariatric populations may provide additional insights. Nevertheless, the interpretation of these findings requires consideration of the variability in surgical procedures included across studies. Additional evidence is derived from studies including patients who underwent various bariatric procedures rather than SG alone. Stoll et al. evaluated tirzepatide in patients with a suboptimal clinical response following metabolic and bariatric surgery and demonstrated significant reductions in body weight together with improvements in metabolic parameters, irrespective of the type of previous surgical procedure. However, these patients should not be considered equivalent to individuals with true postoperative weight recurrence, as insufficient initial weight loss and subsequent weight recurrence represent different clinical scenarios with potentially different underlying mechanisms and treatment goals. Importantly, this study also assessed body composition, highlighting the need to monitor fat-free mass during treatment [38]. Real-world evidence provides additional supportive information regarding the potential effectiveness of tirzepatide in patients experiencing postoperative weight recurrence; however, these data remain limited by methodological constraints. Vinciguerra et al. evaluated 34 patients with recurrent weight gain following bariatric or endoscopic bariatric procedures. After 24 weeks of treatment, mean total weight loss reached 18.1%. Adverse events were predominantly mild gastrointestinal symptoms and did not lead to treatment discontinuation. However, interpretation of these findings is limited by the small sample size, heterogeneous patient population, and inclusion of individuals who had undergone different bariatric procedures rather than SG exclusively [39]. A recent meta-analysis by Osorio Manyari et al. provides additional supportive evidence regarding the potential effectiveness of incretin-based therapies after bariatric surgery. Both semaglutide and tirzepatide were associated with significant postoperative weight loss in patients experiencing recurrent weight gain after MBS. However, the available evidence was derived predominantly from retrospective studies characterized by heterogeneous follow-up periods, varying definitions of postoperative weight recurrence, and methodological limitations [4].

To clarify the hierarchy and applicability of the available post-bariatric evidence, Table 3 distinguishes direct evidence obtained specifically in patients treated with tirzepatide after SG from indirect evidence derived from mixed post-bariatric populations and complementary evidence concerning other incretin-based treatments.

### 6.3. Indirect Evidence from Non-Surgical Obesity Trials

Because direct evidence after SG remains scarce, randomized clinical trials conducted in individuals with obesity without previous bariatric surgery provide important, although indirect, information regarding tirzepatide efficacy, treatment durability, safety profile, and changes in body composition.

The SURMOUNT trials demonstrated substantial weight reduction with tirzepatide; however, these studies did not include patients after metabolic and bariatric surgery. Therefore, these findings may offer indirect support for the biological plausibility and general efficacy of tirzepatide, but they do not constitute direct evidence for postoperative weight recurrence management.

### 6.4. Comparison with Revisional Surgery and Treatment Selection

An important consideration when evaluating tirzepatide is its comparison with revisional bariatric surgery. Revisional surgery remains an alternative therapeutic strategy for selected patients with weight recurrence after SG, particularly when anatomical factors contribute to inadequate clinical response. However, direct comparisons between revisional procedures and pharmacotherapy with tirzepatide remain limited. This observation does not diminish the clinical value of pharmacotherapy but rather emphasizes the importance of individualized treatment selection. Revisional surgery may be more appropriate in patients with anatomical causes of postoperative weight recurrence, whereas tirzepatide represents a valuable non-surgical option for individuals without correctable anatomical abnormalities [26].

### 6.5. Integrated Summary of Evidence

Overall, the currently available evidence suggests that tirzepatide may represent a promising adjunctive therapy for selected patients with postoperative weight recurrence. However, conclusions regarding its role after SG remain limited by the scarcity of direct evidence and rely partly on indirect findings from mixed post-bariatric cohorts and obesity trials.

## 7. Safety of Tirzepatide After Sleeve Gastrectomy

The safety profile of tirzepatide in patients following SG may be evaluated within the context of both the established safety data for the drug and the unique physiological characteristics of the postoperative population. Most available evidence originates from the SURMOUNT clinical trial program, which predominantly included individuals with overweight or obesity who had not undergone previous MBS. Consequently, these findings provide important information regarding the general safety profile of tirzepatide but do not directly establish its safety profile after SG. Extrapolation to patients after SG warrants careful consideration due to altered gastrointestinal anatomy, reduced gastric capacity, variable food tolerance, and the increased risk of nutritional deficiencies [22,41,42].

Gastrointestinal adverse events are the most frequently reported side effects of tirzepatide. In SURMOUNT-1, nausea, diarrhea, and constipation were the most common treatment-emergent adverse events [25]. A similar safety profile was observed in SURMOUNT-2, which included individuals with obesity and type 2 diabetes, where gastrointestinal symptoms—primarily nausea, diarrhea, and vomiting—were again the predominant adverse events [43]. Likewise, in SURMOUNT-3, conducted after an intensive lifestyle intervention, gastrointestinal symptoms were generally mild to moderate in severity and occurred most frequently during dose escalation [26]. Comparable findings have been reported in the SURMOUNT-OSA trial and in several meta-analyses evaluating tirzepatide and other incretin-based therapies [44,45,46].

According to the U.S. Food and Drug Administration (FDA) prescribing information for ZEPBOUND^®^, adverse events reported in at least 5% of treated patients include nausea, diarrhea, vomiting, constipation, abdominal pain, dyspepsia, injection-site reactions, fatigue, hypersensitivity reactions, eructation, alopecia, and gastroesophageal reflux disease [47].

Although gastrointestinal adverse events are usually transient and manageable in the general obesity population, their clinical significance may differ after SG. Reduced gastric volume, altered eating patterns, and limited tolerance of larger meals may increase the consequences of appetite suppression and gastrointestinal symptoms, particularly when they interfere with adequate protein and fluid intake. The FDA warns that tirzepatide has been associated with severe gastrointestinal adverse reactions and is not recommended for patients with severe gastroparesis [47]. In the postoperative SG population, worsening nausea, vomiting, or diarrhea may necessitate clinical evaluation of hydration status, dietary tolerance, and dose escalation strategy, with temporary interruption or postponement of further escalation considered when clinically appropriate. Another important safety consideration is the risk of dehydration and acute kidney injury. According to the FDA, acute kidney injury may occur secondary to dehydration, particularly in patients experiencing prolonged nausea, vomiting, or diarrhea [47]. This risk may be amplified after SG because patients frequently have limited capacity to consume fluids in large volumes. However, the extent to which tirzepatide increases this risk specifically after SG has not been systematically evaluated. Therefore, current recommendations are based mainly on known pharmacological effects of tirzepatide and established principles of postoperative bariatric care rather than direct clinical evidence. Therefore, careful monitoring for dehydration, dizziness, reduced urine output, fatigue, and deterioration in renal function is recommended, particularly in high-risk individuals. Pancreatic and biliary safety also warrants consideration. The FDA has reported cases of acute pancreatitis associated with GLP-1 receptor agonists and tirzepatide, recommending immediate discontinuation of treatment if pancreatitis is suspected [47]. Although the meta-analysis by Zeng et al. did not demonstrate a significant increase in pancreatitis risk, it suggested a possible increase in the composite risk of gallbladder and biliary diseases [46].

This issue is particularly relevant after SG, as rapid postoperative weight loss itself predisposes patients to gallstone formation. Nevertheless, available data do not allow for determination of whether tirzepatide independently increases this risk in patients after SG. Symptoms such as severe abdominal pain, right upper quadrant pain, fever, persistent nausea or vomiting, or pain radiating to the back should be considered potential indications for clinical evaluation. Tirzepatide may also increase the risk of hypoglycemia when administered in combination with insulin or insulin secretagogues, particularly sulfonylureas [47]. In SURMOUNT-2, tirzepatide substantially improved glycemic control and reduced body weight in patients with type 2 diabetes; however, careful adjustment of concomitant antidiabetic therapy was recommended [43]. Among patients after MBS, many of whom experience complete or partial remission of type 2 diabetes, evaluation of hypoglycemia risk may need to be individualized, particularly when irregular meal patterns or variable carbohydrate tolerance are present.

The FDA lists a personal or family history of medullary thyroid carcinoma, multiple endocrine neoplasia syndrome type 2, and severe hypersensitivity to tirzepatide or its excipients as contraindications to treatment [47]. Additional safety considerations include hypersensitivity reactions, progression of diabetic retinopathy in patients with type 2 diabetes, monitoring for depression or suicidal ideation, and the potential risk of pulmonary aspiration during general anesthesia or deep sedation because of delayed gastric emptying [47]. The latter issue may be particularly relevant in patients after MBS who require endoscopic procedures or revisional bariatric surgery.

The limited studies specifically evaluating patients after SG provide preliminary evidence of short-term tolerability; however, these findings should not be interpreted as confirmation of long-term safety. In the retrospective study by Jamal et al., involving patients with postoperative weight recurrence treated with semaglutide or tirzepatide, no serious adverse events were reported during the six-month follow-up period [3]. Similarly, studies including mixed MBS populations suggest acceptable short-term tolerability of tirzepatide; however, interpretation remains limited because these studies combine different surgical procedures and do not provide SG-specific safety conclusions [4,40]. SURMOUNT-4 demonstrated that treatment discontinuation resulted in significant weight regain, whereas continued therapy maintained and further enhanced weight loss [27]. Longer-term follow-up from obesity trials has not identified new major safety signals in populations without previous bariatric surgery [44]. However, these findings cannot establish the long-term safety profile of tirzepatide after SG.

Overall, available evidence suggests that tirzepatide has a generally consistent safety profile in individuals with obesity; however, the safety of its use after SG remains insufficiently characterized. Gastrointestinal adverse events, reduced appetite, and potential effects on fluid and nutrient intake may have greater clinical consequences in postoperative patients. Consequently, management of patients receiving tirzepatide may involve careful monitoring of gastrointestinal symptoms, hydration status, renal function, gallbladder and biliary complications, pancreatitis, hypoglycemia in patients receiving concomitant antidiabetic therapy, endocrine contraindications, and long-term nutritional status. The absence of prospective studies conducted exclusively in patients after SG remains the principal limitation in assessing the long-term safety of tirzepatide in this population.

## 8. Nutritional Considerations of Tirzepatide Therapy After Sleeve Gastrectomy

Direct evidence regarding the impact of tirzepatide on nutritional status in patients after SG is currently lacking. Therefore, nutritional considerations in this population are based on the combination of available post-bariatric nutrition guidelines, general safety data from tirzepatide trials, observations from limited post-bariatric studies, and plausible mechanisms related to appetite suppression and reduced energy intake.

### 8.1. Nutritional Status and Nutritional Deficiencies

Patients after SG remain at risk of nutritional complications despite the absence of a major malabsorptive component. The combined effects of SG and tirzepatide therapy on nutritional status and the risk of nutrition-related deficiencies therefore represent an important clinical consideration. In this population, weight reduction alone may provide an incomplete measure of treatment success, given the clinical relevance of preserving nutritional status and functional outcomes. Patients who have undergone MBS are at risk of inadequate energy and protein intake, reduced tolerance of meal volume and food variety, poor adherence to long-term supplementation, and micronutrient deficiencies. The introduction of a medication that further suppresses appetite and enhances satiety may theoretically contribute to reduced dietary intake in susceptible individuals, particularly when treatment is not accompanied by structured dietetic support [3,6,9,40,48,49,50].

Protein intake is of particular importance. After MBS, patients are generally advised to consume at least 60–80 g of protein per day or approximately 1.1–1.5 g/kg of ideal body weight, with individual adjustment according to clinical status, physical activity, and body composition [50]. Adequate protein intake is essential for limiting the loss of fat-free mass, supporting tissue healing, maintaining immune function and muscle strength, and preserving overall nutritional status. In patients receiving tirzepatide, achieving these targets may be more difficult because of reduced appetite, earlier satiety, nausea, or intolerance of larger meal portions. Therefore, assessment of protein intake may represent an important component of follow-up in patients treated with tirzepatide after SG, although the optimal nutritional monitoring strategy for this specific population has not yet been established [50,51,52,53].

Micronutrient deficiencies represent another important area of concern. Guidelines from the American Society for Metabolic and Bariatric Surgery (ASMBS) and the British Obesity and Metabolic Surgery Society (BOMSS) emphasize the need for lifelong monitoring and supplementation after MBS because patients remain at risk of deficiencies in iron, vitamin B12, folate, thiamine, vitamin D, calcium, and other vitamins and minerals [9,49]. Although SG is less malabsorptive than RYGB, it does not eliminate the risk of nutritional deficiencies. Reduced gastric capacity, lower food intake, food intolerance, irregular supplement use, and persistent gastrointestinal symptoms may all contribute to deterioration in nutritional status after SG [9,49,50]. Tirzepatide therapy may potentially complicate adherence to postoperative nutritional recommendations, particularly in patients experiencing marked appetite suppression or gastrointestinal adverse effects. Appetite suppression, early satiety, and gastrointestinal adverse effects may reduce the consumption of protein-rich foods, vegetables, and foods containing iron, calcium, and B vitamins, while also decreasing adherence to prescribed supplementation. Persistent vomiting is of particular concern because it may increase the risk of dehydration and thiamine deficiency. Accordingly, treatment with tirzepatide after SG may benefit from monitoring of food tolerance, fluid intake, supplement adherence, and symptoms suggestive of nutritional deficiencies, including fatigue, dizziness, paresthesia, impaired concentration, hair loss, angular cheilitis, pallor, and reduced physical capacity.

Hydration is another essential component of clinical monitoring. Patients after SG often have a limited capacity to consume large volumes of fluid at one time, whereas tirzepatide may exacerbate nausea, fullness, or postprandial discomfort. In clinical practice, appetite suppression and gastrointestinal adverse effects associated with tirzepatide may contribute to reduced fluid intake in some patients, particularly those who already have limited drinking capacity after SG. Hydration status may therefore be assessed through evaluation of daily fluid intake, urinary frequency and color, dizziness, constipation, weakness, and, where clinically indicated, electrolyte disturbances [9,49,50].

From this perspective, the limited reporting of nutritional outcomes represents one of the major weaknesses of the current evidence regarding tirzepatide after SG. Most studies focus primarily on body weight, percentage of total weight loss (%TWL), BMI, and metabolic outcomes, while protein intake, dietary quality, supplementation, nutritional deficiencies, food tolerance, hydration, body composition, muscle strength, and the risk of sarcopenia are rarely assessed systematically [3,40]. Consequently, the available data may demonstrate weight-loss efficacy but do not allow for a definitive assessment of whether such weight loss is nutritionally and functionally safe for the patient.

### 8.2. Body Composition and Risk of Sarcopenia

An important consideration in the assessment of pharmacological weight reduction is the quality of body composition changes accompanying weight loss. However, direct evidence regarding the effects of tirzepatide on body composition specifically in patients after SG is currently unavailable. Therefore, current interpretation relies on data from obesity trials, general evidence regarding body composition changes after MBS, and limited post-bariatric pharmacotherapy studies.

Body composition analysis from the SURMOUNT-1 trial demonstrated that tirzepatide treatment resulted in substantial reductions in fat mass; however, these changes were accompanied by a decrease in fat-free mass (FFM) [6,25]. Similar findings have been reported for incretin-based therapies as a class. In a meta-analysis by Karakasis et al., potent incretin-based agents, including tirzepatide and semaglutide, produced marked reductions in both body weight and fat mass but were also associated with decreases in FFM [52]. Although these findings indicate that pharmacological weight loss may involve changes in lean tissue, their applicability to patients after SG remains uncertain because participants in these studies did not represent a post-bariatric population. Therefore, treatment outcomes may benefit from an assessment that extends beyond absolute weight loss alone and considers the quality of weight reduction, including changes in metabolic parameters, functional outcomes, and preservation of lean body mass. The preservation of lean tissue is of particular importance in patients undergoing MBS. A systematic review and meta-analysis by Nuijten et al. demonstrated significant reductions in lean body mass, fat-free mass, and skeletal muscle mass following bariatric surgery, particularly during the first postoperative months [53]. Although loss of adipose tissue is the primary therapeutic goal in obesity treatment, rapid postoperative weight loss may also accelerate the loss of skeletal muscle. Therefore, patients experiencing weight recurrence after SG who undergo renewed pharmacological weight reduction may represent a population in whom preservation of lean mass deserves particular attention. However, whether tirzepatide independently increases the risk of clinically significant muscle loss after SG remains unknown. The limited postoperative evidence available also suggests that body composition should be monitored in addition to total body weight. In a retrospective study, Stoll et al. demonstrated that tirzepatide was associated with clinically meaningful weight loss and improvements in metabolic parameters in patients with suboptimal clinical response after MBS. Importantly, the authors reported differences in FFM loss between patients with weight recurrence and those with insufficient postoperative weight loss, emphasizing that postoperative patients represent a heterogeneous population requiring individualized therapeutic approaches [38,40]. These findings provide important preliminary information; however, the available evidence remains limited by the observational design, heterogeneity of surgical procedures, and absence of a control group. Adequate protein intake remains a cornerstone of nutritional management after MBS, particularly during periods of rapid weight loss. In patients receiving tirzepatide after SG, attention to protein intake and preservation of muscle function may be clinically relevant, although specific nutritional targets for this population have not been established in prospective studies. Clinical follow-up may therefore include assessment not only of body weight but also of protein intake, muscle strength, resistance exercise, and, whenever feasible, body composition using bioelectrical impedance analysis (BIA), dual-energy X-ray absorptiometry (DXA), or other validated assessment methods [6,38,40,49,50]. The potential loss of FFM further supports combining pharmacotherapy with individualized nutritional management and structured physical activity. However, current evidence does not allow for determination of whether such interventions specifically prevent tirzepatide-associated muscle loss after SG. Current evidence suggests that dietary counseling and protein supplementation alone may not be sufficient to fully prevent postoperative muscle loss, while adherence to nutritional recommendations often remains suboptimal [50,52]. Consequently, evaluation of tirzepatide effectiveness after SG may benefit from a comprehensive approach incorporating body composition, muscle strength, protein intake, and resistance exercise alongside conventional anthropometric measures. Such an approach may provide a more clinically meaningful assessment of treatment response while addressing the potential risk of excessive lean tissue loss during weight reduction.

### 8.3. Practical Dietetic Considerations and Clinical Implications

The use of tirzepatide in patients with weight recurrence after sleeve gastrectomy (SG) may have important implications for dietetic practice. However, direct evidence defining optimal nutritional management in this population is currently lacking. Therefore, practical considerations are based on established principles of postoperative bariatric nutrition, available safety data from tirzepatide studies, and potential nutritional consequences of appetite suppression and reduced food intake. Although tirzepatide promotes weight loss by suppressing appetite and enhancing satiety, its use in the postoperative population may require careful nutritional supervision. In addition to weight reduction, preservation of nutritional status, skeletal muscle mass, and development of sustainable long-term dietary habits may represent important components of successful treatment [3,6,23,29,53,54]. Comprehensive nutritional assessment before treatment initiation appears clinically important and may include evaluation of surgical history, postoperative weight trajectory, dietary habits, supplement adherence, and gastrointestinal symptoms. Such an assessment may provide valuable information for determining the most appropriate therapeutic approach, including consideration of pharmacotherapy and optimization of dietary and lifestyle interventions [23,47,49]. A fundamental principle of nutritional management after MBS is the “protein-first” approach. Current guidelines recommend a daily protein intake of at least 60–80 g or approximately 1.1–1.5 g/kg of ideal body weight [20]. Because tirzepatide reduces appetite and promotes early satiety, achieving these recommendations may be challenging. Consequently, patients may benefit from strategies such as small, frequent, protein-rich meals, while protein supplementation may be considered when dietary intake is insufficient [47,49]. Diet quality is equally important. Given the reduced gastric capacity following SG, meals may be planned to emphasize nutrient density while limiting foods with high energy density and low nutritional value, such as sweets, processed snacks, and sugar-sweetened beverages. Gastrointestinal adverse events associated with tirzepatide—including nausea, vomiting, and constipation—may further compromise dietary intake and adherence to nutritional recommendations [22,44,46]. In such cases, individualized dietary modification and assessment of hydration status may be appropriate. Patients should be encouraged to consume fluids regularly in small volumes throughout the day to minimize the risk of dehydration. Long-term micronutrient supplementation and biochemical monitoring remain integral components of postoperative care in accordance with ASMBS and BOMSS recommendations [9,47,49]. Because appetite suppression may further reduce overall nutrient intake, supplementation regimens may benefit from regular review in accordance with established postoperative bariatric recommendations, with adjustments guided by biochemical findings and clinical status. Routine follow-up may incorporate assessment of energy and protein intake, food tolerance, hydration, supplement adherence, biochemical parameters, and body composition. In clinical practice, nutritional priorities may include ensuring adequate protein and fluid intake, followed by emphasis on nutrient-dense foods and adherence to prescribed vitamin and mineral supplementation. Patients presenting with inadequate protein intake, declining muscle strength, persistent gastrointestinal symptoms, or clinical signs of nutritional deficiencies may require dietary intervention and medical reassessment. The dietitian plays a central role in maintaining long-term treatment success. Because weight regain has been observed following discontinuation of tirzepatide in obesity trials [55], nutritional counseling may play a role not only in supporting weight reduction during pharmacotherapy but also in promoting sustainable dietary behaviors that could contribute to long-term weight maintenance. Preservation of skeletal muscle mass may represent an additional clinically relevant consideration.

Because both bariatric surgery and tirzepatide-induced weight loss may contribute to reductions in fat-free mass, body composition should be monitored whenever possible using BIA, DXA, or other validated methods [1,6,52,56]. Resistance exercise represents a fundamental component of muscle preservation. A systematic review by Morales-Marroquin et al. demonstrated that resistance training after MBS may improve muscle strength and, in some patients, attenuate the loss of lean body mass [56]. Accordingly, nutritional management of patients receiving tirzepatide after SG may benefit from combining adequate protein intake, structured resistance exercise, assessment of muscle strength, and body composition monitoring. The overall balance between the potential clinical benefits of tirzepatide therapy, its nutritional risks, and the key aspects of dietetic monitoring after sleeve gastrectomy is summarized in Figure 1.

Clinical assessment before and during tirzepatide therapy may also include evaluation of obesity-related comorbidities and routinely used cardiometabolic parameters, particularly glycemic control, lipid profile, blood pressure, and liver function, according to the individual clinical status.

## 9. Limitations of the Current Evidence and Future Research Directions

Despite a plausible mechanistic rationale and encouraging preliminary findings regarding the potential use of tirzepatide in patients with weight recurrence after SG, the available evidence should be interpreted with caution. The most important limitation of the current literature is the absence of randomized controlled trials conducted exclusively in patients after SG. Existing evidence is derived predominantly from retrospective studies involving relatively small cohorts and short follow-up periods, limiting the assessment of long-term efficacy, safety, and the appropriate clinical positioning of tirzepatide in the management of postoperative weight recurrence after MBS [3,4,37,38,39,40]. Another important limitation is the marked heterogeneity of the available studies. Many investigations combine patients who have undergone different bariatric procedures, including SG and RYGB, as well as individuals with distinct clinical scenarios, such as weight recurrence, partial response, or nonresponse to surgery. Moreover, considerable variability exists in the definitions of postoperative weight outcomes, making comparisons across studies difficult. Consequently, treatment efficacy may be interpreted differently depending on the outcome measure used, including current body weight, body mass index (BMI), weight regained from nadir, percentage of total weight loss (%TWL), or percentage of maximum weight loss regained [3,4]. The effects of tirzepatide on body composition after SG also remain insufficiently investigated. Data from SURMOUNT-1 indicate that weight loss achieved with tirzepatide is accompanied by reductions in fat-free mass [6]. However, no studies conducted specifically in patients after SG have comprehensively evaluated changes in fat mass, fat-free mass, skeletal muscle strength, or the risk of sarcopenia. This represents an important knowledge gap, particularly because preservation of lean tissue is a key determinant of long-term functional outcomes after bariatric surgery. Long-term treatment outcomes following discontinuation of tirzepatide also remain uncertain. In SURMOUNT-4, treatment withdrawal in individuals with obesity resulted in substantial weight regain, whereas continued therapy maintained and further enhanced weight loss [27]. Although these findings were obtained in patients without previous bariatric surgery, they raise important questions regarding postoperative management, including the optimal duration of therapy, long-term adherence, treatment costs, accessibility, chronic safety, and strategies following drug discontinuation. These findings should therefore be interpreted as indirect evidence and cannot be directly applied to patients after SG. Therefore, currently available evidence should be regarded as promising but preliminary, requiring confirmation in prospective, long-term studies specifically involving patients after SG [3,4,9,37,39,55]. From a nutritional perspective, one of the most important evidence gaps is the limited assessment of nutrition-related outcomes. Most studies focus primarily on body weight, BMI, and %TWL, whereas protein intake, overall dietary quality, micronutrient supplementation, nutritional deficiencies, food tolerance, hydration status, and markers of nutritional deterioration are rarely evaluated systematically [3,4,29,37,53]. Given that patients after SG may be particularly vulnerable to nutritional complications, future clinical trials could incorporate comprehensive nutritional assessment alongside body composition analysis and functional outcomes. Such an approach could enable a more complete evaluation of the potential long-term benefits and risks associated with tirzepatide therapy in this specific population. Future randomized controlled trials may further evaluate not only weight-loss efficacy but also body composition, nutritional status, dietary intake, physical function, quality of life, and long-term safety, particularly among patients after SG with clinically defined weight recurrence, using standardized definitions of postoperative weight recurrence. Future prospective studies should also investigate whether changes in routinely assessed metabolic parameters or emerging biomarkers may help predict treatment response, safety, or the recurrence of obesity-related complications after SG.

## 10. Conclusions

Tirzepatide may represent a promising adjunctive pharmacological option for selected patients with weight recurrence after SG; however, current evidence remains limited and should be interpreted with caution. Available evidence suggests that tirzepatide may induce clinically meaningful weight reduction, although direct SG-specific data remain scarce and are based primarily on short-term observational studies. The observed greater efficacy compared with semaglutide in retrospective analyses warrants confirmation through well-designed prospective studies. The potential role of tirzepatide after SG is supported by a plausible mechanistic rationale, as dual GIP/GLP-1 receptor agonism may enhance satiety, suppress appetite, improve glycemic control, and promote weight reduction. Nevertheless, mechanistic plausibility does not replace clinical evidence, and the long-term effectiveness of tirzepatide after SG remains to be established.

Patients following MBS have unique postoperative characteristics, including reduced food tolerance, increased risk of dehydration, nutritional deficiencies, and loss of fat-free mass, which warrant careful clinical consideration. Importantly, treatment outcomes after SG may be assessed beyond body weight, BMI, or percentage of total weight loss alone. Evaluation of treatment response may also incorporate body composition, preservation of skeletal muscle mass, nutritional status, and functional outcomes.

Clinical management may benefit from a multidisciplinary approach involving physicians, dietitians, psychologists or psychodietitians, and physical activity specialists. Before initiating therapy, potential contributors to postoperative weight recurrence, including dietary, behavioral, metabolic, and anatomical factors, may be considered to support appropriate patient selection and individualized treatment planning. Importantly, therapeutic decisions should differentiate between patients with true weight recurrence after an initial successful response and those with insufficient primary weight loss or nonresponse after surgery, as these situations may require different management strategies.

Although currently available evidence is encouraging, it remains limited by the absence of randomized controlled trials, heterogeneous study populations, inconsistent definitions of postoperative weight recurrence, and insufficient assessment of nutritional and body composition outcomes. Future prospective studies may evaluate not only the efficacy of tirzepatide in promoting weight loss but also its long-term effects on body composition, nutritional status, physical function, and the sustainability of treatment outcomes in patients after SG.

## Figures and Tables

**Figure 1:**
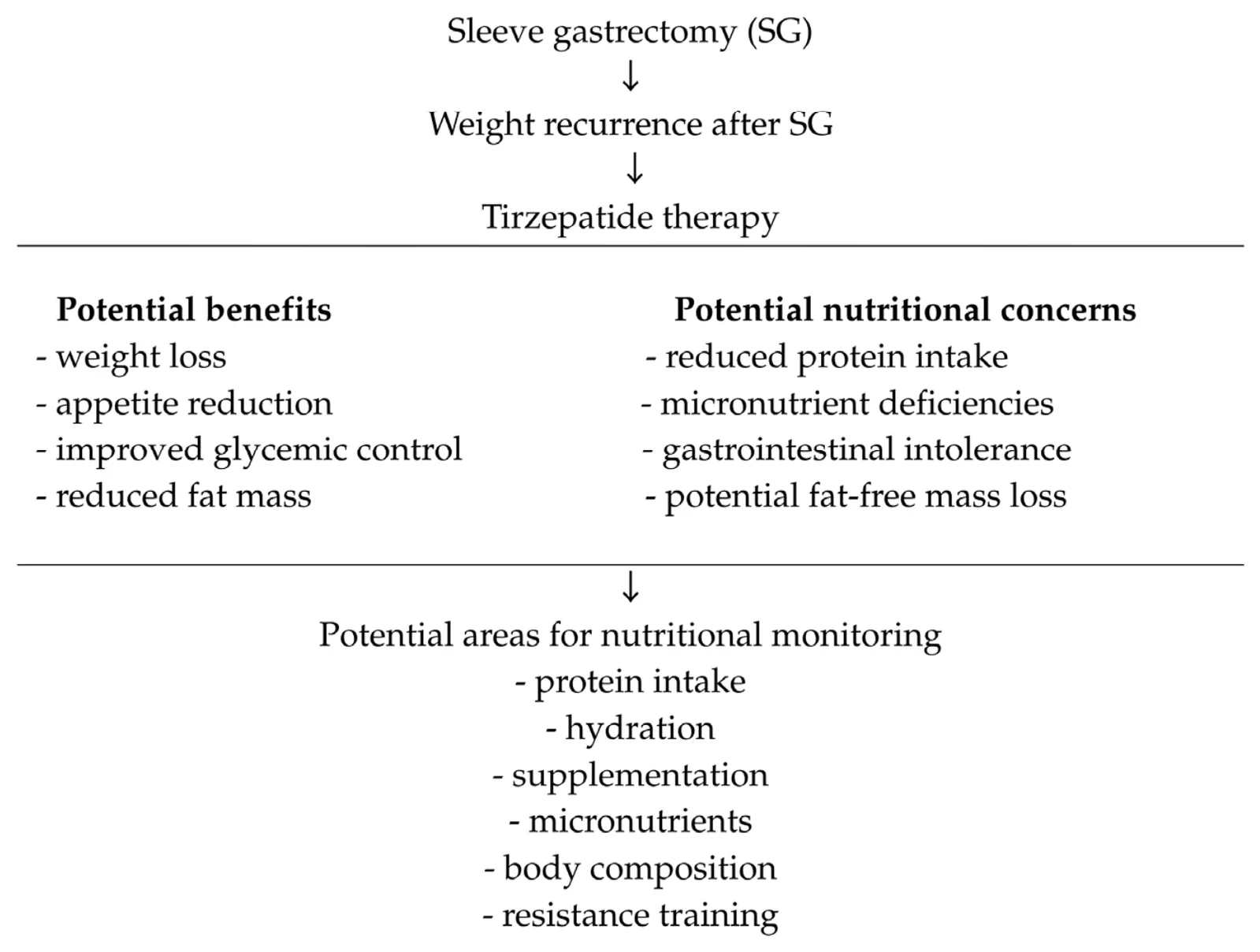
Conceptual Overview of Potential Benefits, Nutritional Considerations, and Monitoring Strategies During Tirzepatide Therapy After Sleeve Gastrectomy.

**Table 1:** Selected Terminology and Outcome Measures Used to Describe Postoperative Weight Outcomes After Metabolic and Bariatric Surgery [1,2,18,20,21,22,23].

Term/Outcome Measure	Definition	Comment
Weight regain	Increase in body weight following initial postoperative weight loss	Widely used but inconsistently defined, may refer to absolute weight gain, BMI increase, weight gain from nadir, or regain of previously lost weight.
Weight recurrence	Recurrence of excess body weight following an initial successful response to bariatric surgery	Preferred terminology because it reflects the chronic, relapsing nature of obesity and avoids patient-blaming language.
Insufficient weight loss/Nonresponse	Failure to achieve an adequate postoperative weight reduction	Differs from weight recurrence because satisfactory weight loss was never achieved.
Partial response	Partial response to bariatric surgery (currently proposed as 10–20% TWL)	Distinguishes patients with moderate weight loss from both nonresponders and those with subsequent weight recurrence.
Suboptimal clinical response	Overall postoperative outcome that fails to achieve or maintain clinically meaningful benefits because of insufficient weight loss, weight recurrence, persistence or recurrence of obesity-related comorbidities, or a combination of these factors	Broad clinical concept encompassing both nonresponse and weight recurrence rather than a single anthropometric measure.
Weight nadir	Lowest body weight achieved after surgery	Essential reference point for evaluating postoperative weight recurrence.
Weight gain from nadir	Difference between current body weight and the postoperative weight nadir	Simple measure but does not account for the magnitude of the initial weight loss.
Percentage of maximum weight loss regained	Percentage of previously lost weight regained after reaching the weight nadir	Demonstrated by King et al. to correlate well with clinical outcomes, Majid et al. proposed >20% as the threshold for clinically significant weight recurrence.
%TWL (or %TBWL)	Percentage of total body weight lost relative to baseline weight	Increasingly preferred because of its simplicity and comparability across studies.
%EWL	Percentage of excess weight loss relative to ideal body weight	Traditionally used in bariatric surgery; however, it depends on the method used to determine ideal body weight.

**Table 2:** Gastrointestinal hormones involved in appetite and satiety regulation after sleeve gastrectomy [28,31,32,33,34,35,36].

Hormone	Primary Site of Secretion	Physiological Role	Changes After SG	Clinical Relevance
Ghrelin	Primarily the gastric fundus	Orexigenic hormone that stimulates appetite, food intake, energy homeostasis, and growth hormone secretion	Fasting ghrelin concentrations generally decrease after SG	A subsequent increase in appetite may indicate that ghrelin suppression alone is insufficient to maintain long-term weight loss.
GLP-1	L cells of the distal small intestine and colon	Incretin hormone that enhances glucose-dependent insulin secretion, suppresses glucagon secretion, delays gastric emptying, and promotes satiety	Postprandial GLP-1 secretion increases markedly after SG	Tirzepatide activates the GLP-1 receptor and may enhance satiety and glycemic control through mechanisms that are biologically relevant after SG.
PYY	L cells of the ileum and colon	Anorexigenic hormone involved in postprandial satiety signaling	Postprandial PYY secretion increases after SG	Increased PYY contributes to satiety, although its effects may diminish over time in patients with weight recurrence.
GIP	K cells of the duodenum and proximal small intestine	Incretin hormone that stimulates glucose-dependent insulin secretion and may influence adipose tissue metabolism	The effects of SG on GIP secretion remain inconsistent	Tirzepatide activates both GIP and GLP-1 receptors, potentially providing complementary metabolic effects in the context of postoperative metabolic adaptations.
CCK	I cells of the duodenum and jejunum	Satiety hormone that stimulates pancreatic enzyme secretion, gallbladder contraction, delays gastric emptying, and enhances satiety	Contributes to postprandial regulation of satiety and digestion	May support satiety after SG but is not a direct pharmacological target of tirzepatide.

**Table 3:** Direct and complementary post-bariatric evidence relevant to the use of tirzepatide after sleeve gastrectomy [3,4,5,38,39,40].

Ref.	Study Design	Population/Previous Bariatric Procedure	Treatment	Main Outcome	Relevance of Evidence
**Direct** **evidence after sleeve gastrectomy**					
Jamal et al., 2024 [3]	Retrospective cohort study	115 patients who had undergone sleeve gastrectomy (SG) with weight recurrence	Semaglutide (*n* = 70) or tirzepatide (*n* = 45); 6 months; 2.5 mg once weekly for tirzepatide and 0.25 mg once weekly for semaglutide	At 6 months, mean weight loss was 10.3 ± 5.9% with semaglutide and 15.5 ± 6.3% with tirzepatide	Its relevance is limited by the retrospective, non-randomized design, short follow-up, and lack of reported nutritional and body-composition outcomes
**Indirect** **evidence from mixed post-bariatric populations**					
Stoll et al., 2025 [38]	Retrospective, single-center observational study; assessments at baseline and after 1, 3, and 6 months	21 adults without T2D with weight recurrence (WR; *n* = 14) or insufficient weight loss (IWL; *n* = 7) after SG (*n* = 11) or RYGB (*n* = 10)	Tirzepatide in addition to autonomous lifestyle modification: 2.5 mg once weekly for the first 4 weeks, followed by 5.0 mg once weekly with further individual dose escalation according to tolerability. At 6 months, doses ranged from 2.5 to 12.5 mg once weekly	At 6 months mean total weight loss was 12.0 ± 3.4%. Fat mass decreased by 9.5 ± 4.3 kg and fat-free mass by 5.5 ± 4.3 kg. Total weight loss and changes in fat and fat-free mass did not differ significantly between SG and RYGB	The study included both SG and RYGB patients, and the primary results were presented for the combined population. Although SG patients comprised 11 of the 21 participants, separate numerical results for efficacy and body composition were not presented for the SG subgroup. Interpretation is further limited by the retrospective design, small sample size, and 6-month follow-up period
Vinciguerra et al., 2026 [39]	Prospective, single-center observational cohort study without a comparator; 24-week follow-up	34 adults without T2D with recurrent weight gain, defined as regain of ≥15% of weight previously lost. Previous procedures: OAGB (*n* = 18), SG (*n* = 13), AGB (*n* = 1), and ESG (*n* = 2)	Once-weekly subcutaneous tirzepatide, initiated at 2.5 mg and increased in 2.5 mg increments according to tolerability, weight-loss response, and patient preference	At 24 weeks, mean %TBWL was 18.1 ± 5.6% in the entire cohort. In the SG subgroup, %TBWL was 18.5 ± 5.4%, compared with 19.0 ± 5.4% after OAGB. Waist circumference decreased by 10.1 ± 3.7 cm (*p* < 0.0001). All patients achieved ≥5% TBWL, 94% achieved ≥10%, 68% achieved ≥15%, and 32% achieved ≥20%	Interpretation is limited by the small SG subgroup, absence of a comparator, non-randomized dose titration, single-center design, and short follow-up
Osorio Manyari et al., 2025 [4]	Systematic review and meta-analysis of eight retrospective studies	964 patients after different MBS	Semaglutide or tirzepatide administered at study-specific doses	Pooled %TBWL was −10.97% with semaglutide and −13.63% with tirzepatide	The tirzepatide estimate was based on a very limited number of retrospective cohorts and did not represent an independent SG-specific population
Brown et al., 2025 [40]	Academic multicenter retrospective comparative study; outcomes assessed from 3 months to 3 years after intervention	4901 adults previously treated with SG because of obesity, subsequently managed for insufficient weight loss or weight recurrence. Patients treated with conversion to RYGB: *n* = 3004; patients receiving a GLP-1 receptor agonist: *n* = 1897	Conversion from SG to RYGB or once-weekly injectable semaglutide or tirzepatide.	Conversion to RYGB was associated with greater weight loss than GLP-1RA treatment at all assessed time points up to 3 years. Reported %TBWL was 26.1% after cRYGB and 13.7% with GLP-1RA treatment	All patients had previously undergone SG, but semaglutide and tirzepatide were analyzed jointly as one GLP-1RA group
Dutta et al., 2024 [5]	Systematic review and meta-analysis of eight studies; protocol registered in PROSPERO (CRD42023473991). Results were reported after 6 and 12 months	557 patients with weight gain or inadequate weight loss after bariatric surgery. The types of previous bariatric procedures were not reported	GLP-1 receptor agonists, specifically liraglutide and semaglutide. Treatment lasted at least 3 months; Doses and titration schedules were not provided	Liraglutide resulted in greater weight loss than placebo at 6 months: MD −6.0 kg. Semaglutide resulted in greater percentage weight loss than liraglutide at 6 months: MD −2.57%, and at 12 months: MD −4.15%. Liraglutide was associated with reductions in fat mass (MD −4.78 kg), lean mass (MD −3.01 kg), and whole body bone mineral density (MD −0.02 kg/m^2^)	The review evaluated liraglutide and semaglutide, not tirzepatide. The population was not restricted to SG, and no SG-specific results were reported

AGB, adjustable gastric band; cRYGB, conversion to Roux-en-Y gastric bypass; ESG, endoscopic sleeve gastroplasty; GLP-1RA, glucagon-like peptide-1 receptor agonist; IWL, insufficient weight loss; MBS, metabolic and bariatric surgery; MD, mean difference; OAGB, one-anastomosis gastric bypass; PROSPERO, International Prospective Register of Systematic Reviews; RYGB, Roux-en-Y gastric bypass; SG, sleeve gastrectomy; T2D, type 2 diabetes; %TBWL, percentage of total body weight loss; WR, weight recurrence.

## Data Availability

No new data were created or analyzed in this study. Data sharing is not applicable to this article.

## Abbreviations

The following abbreviations are used in this manuscript:

ASMBS	American Society for Metabolic and Bariatric Surgery
BIA	Bioelectrical Impedance Analysis
BMI	Body Mass Index
CCK	Cholecystokinin
DXA	Dual-Energy X-ray Absorptiometry
FDA	Food and Drug Administration
FFM	Free Fat Mass
GIP	Glucose-Dependent Insulinotropic Polypeptide
GLP-1	Glucagon-Like Peptide-1
MBS	Metabolic and Bariatric Surgery
PYY	Peptide YY
RYGB	Roux-en-Y Gastric Bypass
SG	Sleeve Gastrectomy
TBWL	Total Body Weight Loss
TWL	Total Weight Loss
